# Integrating tRNA gene epigenomics and expression with codon usage unravels an intricate connection with translatome dynamics in *Trypanosoma cruzi*

**DOI:** 10.1128/mbio.01622-25

**Published:** 2025-08-11

**Authors:** Herbert G. S. Silva, Satoshi Kimura, Pedro L. C. Lima, David S. Pires, Matthew K. Waldor, Julia P. C. da Cunha

**Affiliations:** 1 Laboratório Ciclo Celular, Instituto Butantan 196591, São Paulo, Brazil; 2 Departamento de Microbiologia, Imunologia e Parasitologia, Escola Paulista de Medicina da Universidade Federal de São Paulo 28105, São Paulo, Brazil; 3 Division of Infectious Diseases, Brigham and Women's Hospital 1861, Boston, Massachusetts, USA; 4 Department of Microbiology, Harvard Medical School 1811 , Boston, Massachusetts, USA; 5 Department of Microbiology & Immunology, Cornell University College of Veterinary Medicine 43317, Ithaca, New York, USA; The Ohio State University, Columbus, Ohio, USA

**Keywords:** *Trypanosoma*, codon usage, protein regulation, tRNA, anticodon-codon, codon-anticodon, epigenetics, translatome, gene regulation, posttranscriptional control mechanisms

## Abstract

Codon usage bias impacts protein expression across all kingdoms of life, including trypanosomatids. These protozoa, such as the *Trypanosoma cruzi*, primarily regulate their protein-coding genes through posttranscriptional mechanisms. Here, we integrated codon usage analyses with translatome data to investigate whether codon bias affects translated transcript expression levels in *T. cruzi* life forms. For the first time in trypanosomatids, tRNA sequencing was employed to reveal coadaptation between codon usage and anticodon availability. Despite notable differences in the proteomes of infective and noninfective forms, they exhibited similar pools of tRNAs and similar codon usage preferences, with notable differences in A-site ribosome occupancies. We developed pipelines to measure the adaptation of codons to their corresponding tRNA abundance pool (GM-tECA—Geometric Mean of tRNA Expression-Codon Adaptation) and to calculate the percentage of anticodon:codon base pairing modes in the *T. cruzi* genome. Our pipelines revealed an association between tRNA abundance, anticodon:codon pairing modes, and translated transcript levels. Highly expressed mRNAs are more adapted to tRNA abundance and favor either Watson-Crick or inosine pairing, whereas less expressed mRNAs exhibit lower adaptation to tRNA abundance and enrichment of codon with Wobble (G:U) pairing. Additionally, we observed that open chromatin levels of tRNA genes correlate with tRNA expression in noninfective forms, but not in infective forms, suggesting chromatin states do not control the tRNA pool in the latter. Overall, our findings suggest that protein expression in *T. cruzi* life forms is influenced by a combination of codon usage bias, tRNA abundance, and anticodon:codon pairing modes, but differences in ribosome A-site occupancies between life forms likely reflect additional layers of translation regulation.

**IMPORTANCE:**

Trypanosomatids primarily regulate protein expression at the posttranscriptional level, with codon bias playing a crucial role in controlling protein production across all life forms. This study investigated how codon usage, tRNA abundance, and codon pairing modes influence protein production in *T. cruzi*. Through tRNA sequencing and the integration of epigenomic and translatome data, we discovered that infective and noninfective forms of *T. cruzi* exhibit similar codon usage and tRNA pool preferences, despite having different proteomes. We developed pipelines applicable to any organism to measure codon adaptation to tRNA pools and pairing modes. Our analysis revealed that highly expressed genes are better aligned with more abundant tRNAs and favor Watson-Crick or inosine pairing. These findings suggest an additional layer of gene regulation based on tRNA availability and pairing modes, which impacts protein expression in the different life forms of *T. cruzi*.

## INTRODUCTION

The genetic code, defined by triplet codons in messenger RNAs (mRNAs), dictates the amino acid sequence of proteins. Typically, 64 codons are present in the genome of most organisms, each encoding one of the twenty common amino acids, except for three stop codons (UGA, UAA, and UAG) involved in translation termination (1). Most amino acids are encoded by two to six “synonymous” codons (1). The biased usage of synonymous codons, where a subset of genes has a different codon bias (nonuniform usage of synonymous codons) from that of the remainder of the genome, has been observed among different organisms (2). Such synonymous codon usage bias has been reported to contribute to the optimization of translation and mRNA stability (2–8). However, our understanding of the impacts of codon usage bias on translation remains limited in many organisms.

The tRNAs, which are linked to specific amino acids, have distinct anticodon sequences that bind to the triplet codons in mRNA, thereby translating the genetic code into amino acids in the A site of ribosomes (9, 10). The correspondences between tRNA anticodons and mRNA codons are complex. The pairing of the anticodon-codon sequences could be mediated by different interactions, such as Watson-Crick and wobble base pairing. The Watson-Crick interaction demands a precise match of the triplet, following the strict rules of complementary pairing (11, 12). On the other hand, wobble base pairing via inosine modification or G:U interactions allows flexibility at the first position of the anticodon (5′−3′) to recognize some ribonucleotides at the third position of the codon (5′−3′). For instance, inosine bases can pair with A, U, or C in codon sequences, while G pairs with U in the anticodon:codon interaction context (13–15). Thus, in many cases, a tRNA binds to multiple synonymous codons, and a single codon can be decoded by multiple tRNA species. Such redundancy in codon:anticodon pairing enables balanced and robust codon decoding.

The balance between codon usage patterns and the availability of tRNAs is crucial for efficient translation (2, 3, 16–18). Matching the supply of tRNAs to the demand for codons is necessary for rapid and constant rates of translation. A low availability of tRNA leads to reduced rates of translation and ribosome pausing, which leads to reduced protein production and sometimes triggers stress responses (8, 19).

The tRNA abundance is regulated through genetic, epigenetic, and posttranscriptional mechanisms, including tRNA modifications (20–24). It has been reported that tRNA pools can be adjusted in response to different environmental cues, enabling rapid and increased synthesis of proteins essential for cellular adaptation to environmental changes (25). Substantial changes in the expression of different tRNA isodecoders are observed in different mouse tissues (26) and during the differentiation of human cells (27), although the overall tRNA anticodon pools (related to the decoding rates) are largely stable. Therefore, profiling the dynamics of tRNA abundance is critical for understanding the impact of codon usage bias on protein synthesis.

Several methods have been used to estimate tRNA abundance profiles. tRNA genomic copy numbers have been widely used since, in many eukaryotes, they correlate with the abundance of their corresponding codons (28–30). Although they are widely used, it is not clear whether they reflect the actual tRNA expression profiles in most organisms or under different conditions. The recent development of tRNA sequencing has enabled direct profiling of tRNA expression, but this technique has been applied only to few organisms and under a limited number of conditions. Direct profiling of tRNA abundance and its correlation with codon usage bias is crucial for understanding the principles of global translation regulation.

Trypanosomatids are a group of unicellular parasitic protozoa that include pathogens such as *Trypanosoma cruzi* (*T. cruzi*), *Trypanosoma brucei* (*T. brucei*), and *Leishmania major* (*L. major*). These protozoa exhibit polycistronic transcription of mRNA genes, and their gene expression regulation occurs mainly posttranscriptionally (31). *T. cruzi* is the etiological agent of Chagas disease and has four different developmental stages: two infective and nonproliferative stages (metacyclic trypomastigotes—MTs and tissue culture trypomastigotes—TCTs) and two noninfective and proliferative stages (epimastigotes—EPIs and amastigotes) (32). *T. cruzi* differentiation from EPIs to MTs is followed by changes in gene expression, which is important for the parasite’s virulence and survival in its new habitat (33–35).

EPIs predominantly translate housekeeping genes, while MTs have increased production of virulence-factor proteins (33, 35). Codon bias has been proposed as an important mechanism to direct global relative mRNA and protein expression levels in trypanosomatids (6, 36). However, a poor correlation between codon usage frequency and the copy number of tRNA genes (tDNAs) was observed in *T. cruzi* (37), suggesting that tRNA abundance is controlled by additional mechanism(s). In addition, how codon usage and tRNA profiles affect the *T. cruzi* translatome and development is largely unclear.

Here, we combined multiple genome-wide data sets to investigate the relationships among tRNA abundance, codon usage, gene expression profiles, and tRNA epigenomics in two life forms of *T. cruzi*. For the first time in a trypanosomatid, we employed tRNA sequencing to profile tRNA abundance, associating it to distinct chromatin states and transcript translated levels. Additionally, we developed the GM-tECA (Geometric Mean of tRNA Expression-Codon Adaptation) pipeline, which provides a quantitative measure of the adaptation of mRNAs to the availability of their corresponding tRNA pool. In general, our analyses revealed a previously undescribed layer of gene expression regulation in the life forms of *T. cruzi*, based on codon bias, tRNA abundance, and anticodon-codon base pairing.

## MATERIALS AND METHODS

### Cell culture

*T. cruzi* (Dm28c strain) EPIs were cultivated at 28°C in Liver Infusion Tryptose (LIT) medium supplemented with 10% fetal bovine serum (FBS; Vitrocell), 0.4% glucose, 0.1 µM hemin, and 60 mg/mL penicillin G, as described by (38, 38). The metacyclic trypomastigote forms were obtained following the protocol outlined by (39, 39) with some modifications. In brief, epimastigotes in the exponential growth phase (4 × 10^6^ parasites/mL) were cultured for 4 days until they reached the stationary phase (5–6 × 10**^7^** parasites/mL). Subsequently, the parasites were resuspended at a concentration of 5 × 10^8^ parasites/mL in Triatomine Artificial Urine (TAU) medium (190 mM NaCl, 17 mM KCl, 2 mM CaCl_2_, 2 mM MgCl_2_, and 8 mM phosphate buffer, pH 6.0) and incubated for 2 hours at 28°C. Then, the parasites were diluted to 5 × 10^8^/mL in TAU 3AAG medium (containing 10 mM L-proline, 50 mM L-glutamate, 2 mM L-aspartate, and 10 mM glucose) and maintained in a CO_2_ incubator at 28°C. Metacyclic forms of the *T. cruzi* Dm28c strain were harvested from the culture supernatant after 76 hours of incubation and purified using DEAE-cellulose resin (Sc-211213).

### tRNA species identification

The *T. cruzi* Dm28c (version 42-2018) genome and its coordinates were downloaded in FASTA and gff formats, respectively, from the TritrypDB platform (https://tritrypdb.org/tritrypdb/app/downloads). To obtain the list of tDNAs and their respective coordinates, the term “tRNA” was used as a filter in the gff file. Identification of selenocysteine tRNA (tRNA^Sec^) was performed using the BLASTp tool v.2.10.0 (40) to search for the best hit via the TriTrypDB platform (https://tritrypdb.org/tritrypdb/app/workspace/blast/new). The query reference was “tRNA selenocysteine” (ID = Tb927.9.2380) from *T. brucei* TREU927. tRNAscan-SE software (http://lowelab.ucsc.edu/tRNAscan-SE/) (41) was used to identify the transcribed sense strands and anticodons of the tDNAs. Afterward, a genome FASTA file containing all identified tDNA sequences was obtained. 

#### tRNA sequencing

Total RNA was extracted using TRIzol (Invitrogen) following the manufacturer’s instructions. tRNA purification and sequencing were performed according to (42, 42) with some modifications. Five micrograms of RNA samples was run on a 10% denaturing gel (TBE-UREA), and bands corresponding to tRNAs (70 to 85 bp) were excised, digested, eluted, and recovered by isopropanol precipitation. Subsequently, 250 ng of purified tRNA was deacetylated with 500 µL of 100 mM Tris-HCl (pH 9.0) solution, incubated for 1 hour at 37°C, and recovered using isopropanol. For the adapter-index ligation to tRNA 3′ end, RNase inhibitor (Applied Biosystems/Life Technologies #N8080119) was added with incubation at 80°C for 2 minutes, followed by cooling on ice. Next, 12 µL of PEG mix buffer (10 µL of 50% PEG8000—NEB #B1004 and 2 µL of RNA ligase buffer—NEB #B0216S), 3 µL of the adenylated adapter-index sequence (33 pmol/µL), and 1 µL of T4 RNA ligase (NEB #M0242S) were added, followed by incubation at 25°C for 2.5 hours. The adapter-index sequences are available in (42, 42). The tRNA adapter-index linked (approximately 120 bp) was purified on a denaturing gel. The cDNAs were synthesized with 5 pmol of TGIRT-III (InGex #TGIRT10) in 100 mM Tris-HCl (pH 7.5), 0.5 mM EDTA, 450 mM NaCl, 5 mM MgCl₂, 5 mM DTT, 1 mM dNTPs, and 1.25 pmol of the primer, followed by incubation at 60°C for 1 hour. The primer sequence used for the reverse transcription reaction was as follows: /5Phos/AGATCGGAAGAGCGTCGTGTAGGGAAAGAGTGT/iSp18/CAAGCAGAAGACGGCATACGAGATCG. Subsequently, cDNAs were purified by denaturing gel electrophoresis to isolate fragments of approximately 140 bp. Circularization was performed using 50 U of CircLigase II ssDNA ligase (Epicenter/Lucigen #CL9021K) at 60°C for 1 h, with an additional enzyme addition and incubation cycle to ensure complete circularization. Circularized cDNAs were amplified using Phusion High-Fidelity DNA Polymerase (NEB #M0530S) using the following condition: 98°C for 30 sec; (8×: 98°C for 10 sec, 60°C for 10 sec, and 72°C for five sec). The reverse primer used was CAAGCAGAAGACGGCATACG in all reactions, while index-specific forward primers were as described in 42, 42. The cDNAs purified on a denaturing gel were sequenced in biological duplicates for EPIs and MTs on the Illumina NextSeq 1000 system (single-end). The removal of adapters (AGATCGGAAG) from the FASTQ files was performed using cutadapt v.3.5 (43), followed by HEADCROP:2 using the TrimmomaticSE v.0.39 tool (44). The tDNA FASTA file generated in this study (see Table S1A) was used for mapping. The (i) mapping, (ii) abundance counting, (iii) analysis of differential tRNA expression, and (iv) counting of 3′CCA on tRNA and misincorporation frequencies between epimastigote and trypomastigote metacyclic forms were performed using the mim-tRNAseq script with the parameters --cluster-id 0.97, --max-mismatches 0.075, and --max-multi 4 (45), executed in Python 3.9.18 and R 4.3.1. Only unique mapped reads were aligned to the *T. cruzi* tDNA reference sequences to determine tRNA abundance values. The tRNA-seq data set was deposited at PRJNA1124437. The percentage of misincorporation in the A34 position on tRNAs from EPI and MT forms was determined according to (42, 42). The samples from EPI or MT forms were analyzed in biological duplicates.

### Identification of RNA polymerase III subunits in the *T. cruzi* Dm28c genome

The sequences of the RNA polymerase III subunits (RPC1, RPC2, RPC40, RPC19, RPB6, RPB5, RPB8, RPB10, RPC10, RPC11, RPC17, RPC25, RPC82, RPC53, RPC37, RPC34, and RPC31) of *Saccharomyces cerevisiae* S288c were downloaded from the FungiDB database (https://fungidb.org/fungidb/app). Subsequently, two approaches were employed to identify orthologs in the *T. cruzi* Dm28c strain (version 42-2018) genome. The first approach involved using the BLASTp tool v.2.10.0 (40), which is available on the TriTrypDB platform (https://tritrypdb.org/tritrypdb/app/) to search for alignments with an E-value ≤0.001, followed by confirmation of the protein domain using the platform (https://www.ebi.ac.uk/interpro/search/sequence/). For the unidentified RPC31 protein homologs using the BLASTp method, probabilistic models were created with the HMMER Algorithm v.3.3 (46) based on orthologous sequences of RPC31 from several eukaryotes. Subsequently, a search for the best hits was conducted within the *T. cruzi* Dm28c strain (version 42-2018) genome using the Ubuntu v.20.04 system.

### FAIRE-seq, MNAse-seq, modified base analysis in *T. cruzi* life forms

The RPGC values available from FAIRE-seq data performed in the EPI and MT forms were obtained from (47, 48). Raw files from MNase-seq data in the EPI and TCT forms were obtained from the SRA project number PRJNA665060 (48). The reads were processed using Trimmomatic (44) to remove adapter sequences, and the following parameters were applied: HEADCROP:5, SLIDINGWINDOW:15:25, and MINLEN:35. The rRNA reads were masked using bedtools maskfasta v2.31.1. Read mapping was performed using Bowtie2 (49) to the *T. cruzi* Dm28c genome (version 42-2018) available on TriTrypDB using the following parameters: -D 25, -R 4, -N 0, -L 19, and -i S,1,0.40. SAM files were converted to BAM files, which were subsequently sorted and indexed using SAMtools version 1.12 (50). Read count values for tDNA regions, selenocysteine loci, and shuffled regions equivalent to tRNAs (generated by running Bedtools v2.31.1 with the shuffle option) were obtained using BAMscale Version v1.0 (51). The resulting counts in FPKM were analyzed using libraries such as readr, dplyr, reshape2, ggpubr, and ggplot2 in R Version 4.1.2. Modified base analysis was performed as described in (52, 52). In short, public Oxford Nanopore sequencing data sets available at (53, 53) from EPI and MT forms were converted into POD5 files using pod5 library version 0.3.6 (pod5 convert fast5), which were used to call methylation (m) and hydroxymethylation (h) cytosines with the Dorado program, version 0.5.3, followed by read mapping using the minimap2 program (54) against the genome of the Dm28c 2018 strain (version 60). The analysis of methylated bases was performed using the Modkit program, version 0.2.5 (available at https://github.com/nanoporetech/modkit).

### Ribo-seq and RNA-seq data analysis, A-site ribosome occupancy, and estimation of translational efficiency (TE)

Ribo-seq and RNA-seq data for EPI and MT forms of *T. cruzi* were obtained from the Sequence Read Archive (SRA) repository, project ID: PRJNA260933, provided by 33. The FASTQ files were filtered using the TrimmomaticSE v.0.39 tool (44) with parameters –HEADCROP:1 and MAXINFO:24:0:0.05 and mapped to the *T. cruzi* Dm28c (version 42-2018) genome using Kallisto’s algorithm to obtain the transcripts per million (TPM) values with the following settings: -b 100–l 50–s 20 (55). The DESeq tool v.1.42 (56) was used to perform differential expression analysis of the translatome between EPI and MT forms. To calculate the codon occupancy on site A of the ribosome from EPI and MT forms, the Ribo-seq trimmed reads were mapped using bowtie2 (49), and BAM files were obtained using samtools (50). The BEDTools v.2.26.0 (57) was used to quantify the number of aligned reads overlapping in the *T. cruzi* genome using the functions -wa and -wo (intersect). The distance between the 3′ end of the reads and the A-site position, estimated to be 23 nucleotides, was determined from the highest read count in the distribution of 3′ end reads. The read count for all codons at the A site in EPI and MT forms was divided by 1,000 to obtain their relative frequencies. Translational efficiency (TE) was calculated using the Ribodiff tool (58) with the following parameters: -e and -c. The raw data, including RNA-seq and Ribo-seq data sets from EPI and MT, were previously obtained using Kallisto, as described above, and were used as input files.

### Codon usage analysis in the *T. cruzi* Dm28c genome

The aa.usage.py script available at https://github.com/rhondene/Codon-Usage-in-Python was used to compute codon relative adaptive weights and the absolute codon frequencies in FASTA files. All upregulated CDSs between EPIs (1,752 genes) and MTs (361 genes) were generated from Ribo-seq data, excluding pseudogenes. The reference set was selected based on genes with a log2-fold change of ≤2 (EPIs vs MTsMTs vs EPIs) and an adjusted *P* value of ≤ 0.05, excluding pseudogenes and genes present in more than one copy. Transcripts with >2 fold differential expression are likely regulated by *cis*-acting RNA-binding proteins regardless of codon bias. Pseudogenes were excluded because they may represent spurious open reading frames that may not encode functional transcripts. Lastly, genes with more than one copy were omitted because they compromise accurate TPM calculation and may introduce mapping errors. The top 100 and bottom 100 genes (from the reference set) were obtained from the Ribo-seq data containing the CDSs with 100 genes with higher or lower TPM values in the EPI and MT forms. IDs and TPM abundance for the above mentioned gene sets are available at Table S3. The tRNA abundances, represented as the proportion of mapped reads, were determined for EPIs and MTs. To refine these measurements, a renormalization process was applied to address the potential for a single tRNA to recognize multiple codons. This was achieved by dividing the abundance of each tRNA by the number of its corresponding codons and then assigning these adjusted abundance values to each codon. For codons recognized by multiple tRNA species, the total abundance was calculated by summing the adjusted abundances of all tRNA species recognizing that codon.

### GM-tECA and percentage of anticodon:codon base pairing

The GM-tECA and percentage of anticodon:codon base pairing values were obtained using the custom R script available in https://github.com/trypchromics/trnaExpCodAdapt. First, the sapply function in R 4.3.1 was used to map each codon from the *T. cruzi* (Dm28c) CDSs nucleotide sequence to its corresponding abundance value from the tRNA expression data. The geometric mean of codon values for each CDS in a FASTA file was calculated using the custom function geom_avg_codons in R, based on the input codon table to calculate the GM-tECA values. The table included 61 codon triplets with corresponding values for tRNA abundance, renormalized according to the item “Codon usage analysis in the *T. cruzi* Dm28c genome” and multiplied by 100 to avoid numbers very close to zero. The following formula was used: $\sqrt[{n}]{\prod_{i=1}^{n}\left(100\times {\text{codons}}_{i}\right)}$, where *n* = 61. To calculate the percentage of target anticodon:codon pairing modes, we used a table containing all the same 61 codon triplets. For each codon corresponding to the specific pairing mode, a value of 1 was assigned instead of tRNA abundance. All other codons, which were not targets of this pairing, were assigned a value of 0. Subsequently, the arithmetic mean of the codon values for each CDS was calculated using the arithm_avg_codons function in R, rather than the geometric mean. These arithmetic mean values represent the percentage of the target pairing mode for each sequence.

### Statistical analysis

R (v. 4.3.1) was used to calculate the Spearman and Pearson correlations. Pearson’s correlation was applied to data with normal distribution and no significant outliers (≤5%). Spearman’s correlation was used for data containing non-normal distribution or outliers exceeding 5% of the data. Wilcoxon–Mann‒Whitney and one-way ANOVA tests were calculated using Prism (v. 8.0.1).

## RESULTS

### Characterization of tRNA isotypes and anticodon:codon pairing modes

**Fig 1:**
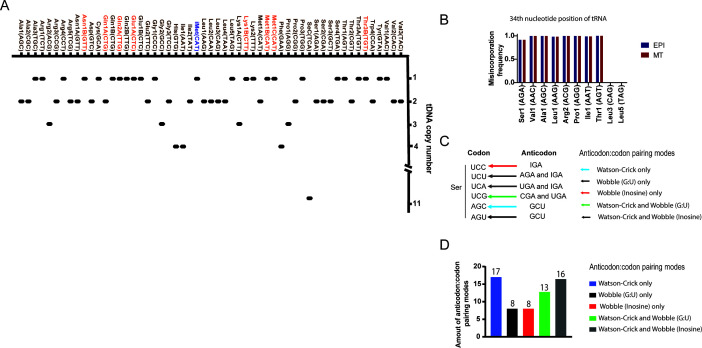
Identification of tRNA isotypes and anticodon:codon base pairing modes in the *T. cruzi* (Dm28c) genome. (**A**) tDNA copy number for all tRNA species in the *T. cruzi* genome. The tRNA isoacceptors (main member) and isodecoders are represented by brown and orange, respectively. tRNA^imet(CAT)^ is represented in blue. (**B**) RT signature (misincorporation frequency) for inosine modification at the 34-nucleotide position of tRNAs in the EPI and MT forms obtained from tRNA-seq data. tRNA^Leu3 (CAG)^ and tRNA^Leu5 (TAG)^ were used as negative controls for the absence of this modification. (**C**) Illustration of the anticodon:codon base pairing modes found in the *T. cruzi* genome. (D) Number of anticodon:codon pairing modes [(i) Watson–Crick only, (ii) wobble (G:U) only, (iii) wobble (inosine) only, (iv) Watson–Crick and wobble (G:U), and (v) Watson–Crick and wobble (inosine)].

The genome of the hybrid *T. cruzi* strain CL Brener (reference strain) encodes 46 anticodons, allowing for the translation of 62 codons representing the 20 canonical amino acids and one noncanonical amino acid, selenocysteine (Sec) (37). The nonhybrid *T. cruzi* strain Dm28c displays a lower tDNA copy number, with a total of 105 tDNAs compared to 120 copies found in the CL Brener strain (37, 59). Our study focused on the *T. cruzi* Dm28c strain, which, similar to the CL Brener strain, has poorly characterized anticodon:codon pairing modes. Thus, we used the tRNAscan-SE tool to characterize the isoacceptors and isodecoders and subsequently established their respective base pairings with the 61 codons that encode the canonical amino acids present in the *T. cruzi* Dm28c genome. Isoacceptors carry the same amino acid despite differences in their anticodon sequences, whereas isodecoders share identical anticodon sequences but exhibit differences in the remainder of their tRNA sequences. The number of isodecoders in the genome excludes the most highly represented tRNA member (60). We identified 105 tRNA genes with 46 anticodons in the *T. cruzi* Dm28c strain (Fig. 1A; Table S1A) that specifically carry one of twenty canonical amino acids found in nature, as well as one tRNA that carries the noncanonical amino acid selenocysteine (tRNASec(TCA)). The majority of tRNA species are present as single- or two-gene copies, except for tRNA^Sec(TCA)^ that has eleven copies located in tandem at PRFA01000024, as depicted in Fig. 1A.

In addition, we evaluated whether tRNA anticodons containing adenosine at the first position (5′−3′) could undergo modification to inosine (I) in the EPI and MT forms as a strategy to complement codons with missing anticodon matches. The I modification provides wobble base pairing flexibility between synonymous codons. For instance, tRNA^Ala(CGA)^ modified to tRNA^Ala(CGI)^ recognizes codons ending (5′−3′) with U, C, and A as Ala (GC**U**), Ala (GC**C**), and Ala (GC**A**), respectively, expanding its recognition beyond Ala (GCU). The detection of I can be achieved using tRNA-seq data as this modification induces high levels of nucleotide misincorporation (90% to 100%) at modified base positions during reverse transcriptase (RT) from RNA to cDNA, thereby generating an RT signature (42, 61). Thus, we sequenced the total tRNAs from both the EPI and MT forms in biological duplicates, and the reads were mapped onto a FASTA file genome composed of 55 tRNA sequences (Fig. S1). The sequencing reads exhibited high quality, coverage, and reproducibility between the duplicates (Fig. S1B to D). We found that all tRNA anticodons beginning (5′−3′) with adenosine in *T. cruzi* are modified by inosine, including tRNA^Ser1 (AGA)^, tRNA^Val1 (AAC)^, tRNA^Ala1 (AGC)^, tRNA^Leu1 (AAG)^, tRNA^Arg2 (ACG)^, tRNA^Pro1 (AGG)^, tRNA^Ile1 (AAT)^, and tRNA^Thr1 (AGT)^. To investigate whether I34 levels vary between non-infective and infective forms, we quantified I34 based on misincorporation frequencies observed in the tRNA-seq data set. We found that these modifications remain stable when comparing the EPI and MT forms, showing no detectable changes in their levels across both life forms (Fig. 1B).

**Fig 2:**
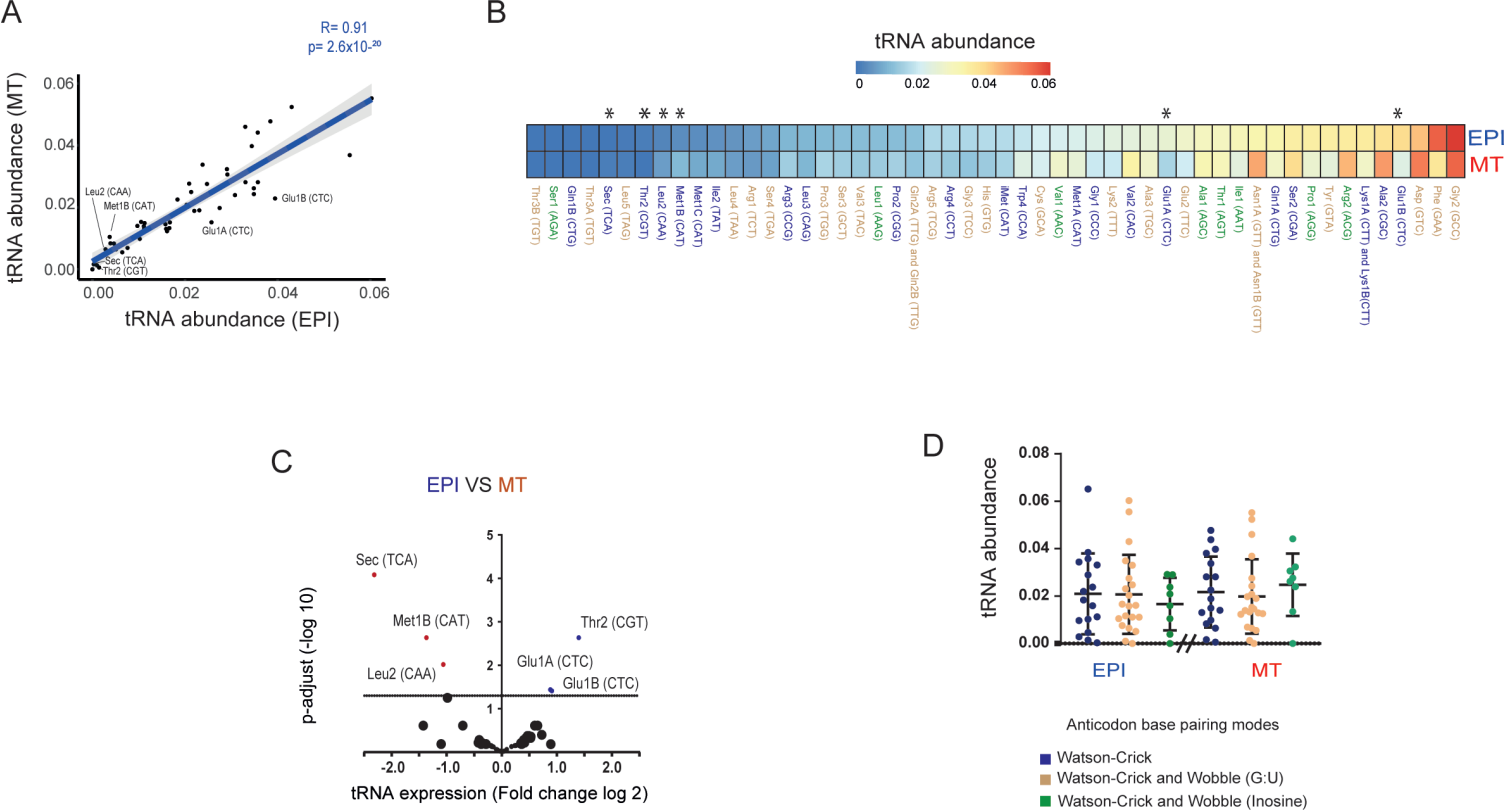
Abundance of tRNA transcripts in EPIs and MTs. (**A**) Scatter plot showing Pearson’s correlation between tRNA abundance (proportion of mapped reads) in the EPI and MT forms. Gray shading, 95% CI. (**B**) Heatmap with tRNA abundance (average of two biological replicates from each life form) levels normalized by the proportion of mapped reads on the tDNA genome from *T. cruzi*. The tRNA anticodons that exhibit only Watson–Crick base pairing with synonymous codons are highlighted in blue. The tRNA anticodons containing either Watson–Crick or wobble base pairing are represented by orange, while those interactions of either Watson–Crick or inosine are illustrated in green.* means tRNAs with statistically significant differences, as detected in C. (**C**) Volcano plot of fold changes of tRNA expression (log2) (*n* = 52) in EPIs compared to MTs. The significance of tRNA differential expression was determined using the DESeq2 algorithm considering a p-adjust value of ≤0.05. (D) Abundance of tRNA species grouped according to their type of base interaction [(i) Watson–Crick, (ii) Watson–Crick and wobble (G:U), and (iii) Watson–Crick and wobble (inosine)] with their corresponding codons. Statistically significant tests (*P* value ≤ 0.05) were performed with the Wilcoxon–Mann‒Whitney test (for *P* values: * =0.05; ns = not significant). tRNA abundance was calculated as the average of two biological replicates per life form. The individual tRNA abundance values for each replicate are shown in Fig. S2A to B. All analyses were performed considering all tRNA species. Isoacceptor analysis is shown in Fig. S2C.

Based on the variations of anticodons in tRNA genes, we depicted all anticodon:codon base pairing possibilities in *T. cruzi* [(i) Watson–Crick only, (ii) wobble (G:U) only, (iii) wobble (inosine) only, (iv) Watson–Crick and wobble (G:U), and (v) Watson–Crick and wobble (inosine)] (Fig. 1C), in which 17 codons display only Watson–Crick pairing with their corresponding tRNA anticodon, 8 display either wobble (G:U) or wobble (inosine) pairing, 13 display Watson–Crick and wobble (G:U) pairing, and 16 codons are recognized by Watson–Crick and wobble (inosine) (Fig. 1D and Table S1A and S1B). The potential implications of the observed anticodon–codon distribution will be addressed in a later section.

### The relative abundance of tRNAs is similar in the EPI and MT forms

The global quantification of tRNA species through sequencing has significant challenges, primarily due to the presence of numerous nucleotide modifications in tRNA molecules. These modifications can lead to either nucleotide misincorporation or incomplete conversion of RNA to cDNA during library construction, leading to challenges in mapping and quantification (45). Additionally, the presence of multiple isodecoders, which contain variations in tRNA sequences outside of the anticodon, makes accurate mapping difficult. To circumvent these challenges, we used a highly processive reverse transcriptase (TGIRT-III), which enables complete cDNA synthesis of modified RNAs and the mim-tRNAseq analysis pipeline (42, 45). We found that the EPI and MT forms of *T. cruzi* displayed a very strong positive correlation (Pearson’s *R*  = 0.91) related to their tRNA abundance (Fig. 2A). Three [(i) Asn1A (GTT)/Asn1B (GTT); (ii) Lys1A (CTT)/Lys1B (CTT); (iii) Gln2A (TTG)/Gln2B (TTG)] (Fig. 2B; Fig. S2B and Table S1C) of the eight isodecoders could not be definitively mapped to their isoacceptors due to the high similarity between these sequences and the lack of unique mapped reads.

EPIs and MTs express different sets of genes. In particular, EPIs mainly express genes encoding housekeeping proteins, whereas MT exhibits increased expression of virulence proteins (33, 35). It has been shown that some organisms readjust their tRNA pool to meet the demands of proteins with increased expression in response to changing environmental conditions (25). Since EPIs and MTs inhabit different environments and exhibit changes in protein expression, we questioned whether there would be a readjustment in tRNA abundance across the different life forms. We observed that the tRNA pool is minimally modulated across *T. cruzi* life forms (Fig. 2C; Fig. S2C and Table S1D). Only six (tRNA^Sec(TCA)^, tRNA^Met1B(CAT)^, tRNA^Leu2(CAA)^, tRNA^Glu1A(CTC)^, tRNA^Glu1B(CTC)^, and tRNA^Thr2(CGT)^) out of 55 tRNA species exhibit expression differences between EPI and MT forms, suggesting the readjustment of the tRNA pool is not necessary to meet codon demands in both life forms of *T. cruzi*.

Furthermore, we found that tRNAs with anticodons exhibiting wobble base pairing are not more abundant than those relying solely on Watson-Crick base pairing. This suggests the lack of a regulatory mechanism to increase the abundance of wobble tRNAs to meet the demand for multiple codons (Fig. 2D and Table S1E). We also evaluated whether tRNAs located in genomic clusters or found as single-copy genes showed differences in expression. No significant difference was detected between these two groups (Fig. S2D). Among the clustered tRNAs, the coefficient of variation (CV) in tRNA abundance within each cluster ranged from 0.64% to 90.8%. Differences were also observed between life forms: for example, for the tRNAs located at PRFA01000070 (65,964–66,197), the CV was 32.2% in EPIs but only 0.64% MTs. While our results indicate some variability in tRNA abundance within clusters, the small number of clusters analyzed (*n* = 3) limits statistical analysis and therefore our ability to draw definitive conclusions (data not shown).

### The chromatin profile of tDNAs correlates with the abundance of their transcripts in the epimastigote form

**Fig 3:**
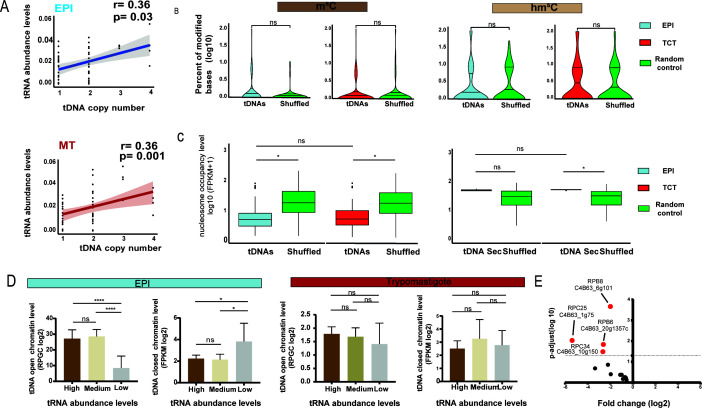
Correlation between tDNA chromatin and tRNA abundance in *T. cruzi* life forms. (**A**) Spearman’s correlation between tRNA abundance and the number of corresponding tDNA copies in the EPI and MT forms. tRNA^Sec^ is not included in the scatter plot. Red or blue shading, 95% CI. (**B**) Percentage of m^5^C and hm^5^C modifications on tDNA nucleotides present in the EPI and TCT forms. log₁₀(100) =2, corresponding to 100% of modified bases. Statistical significance tests were performed with the Wilcoxon–Mann‒Whitney test. (**C**) Nucleosome enrichment in tDNAs and random controls (shuffled) in the EPI and TCT forms using fragments per kilobase per million mapped reads (FPKM) +1 (log10) values from MNase-seq data. Shuffled was used as random genomic controls. Statistical significance tests were performed with the Wilcoxon–Mann‒Whitney test. (**D**) Bar chart showing the associations between high-, medium-, or low-abundance tRNAs and their corresponding tDNA chromatin profiles. The open chromatin levels were normalized to the reads per genomic content (RPGC) from FAIRE-seq data obtained with the EPI and MT forms. The closed chromatin was normalized to the FPKM values from MNAse-seq data obtained with the EPI and TCT forms. Statistical significance tests were performed with the Wilcoxon–Mann‒Whitney test. (**E**) Volcano plot depicting the fold changes and *P* values of RNAP III subunits in *T. cruzi* life forms (EPI vs MT) using Ribo-seq data and DESeq2 algorithm. A *P* value of ≤ 0.05 and fold change ≥1.5 were considered statistically significant. tRNA abundance was calculated as the average of two biological replicates per life form. *P* values: **** =0.0001; * =0.05; ns = not significant.

The tDNA copy number exhibits a strong positive correlation with their transcript abundance in some eukaryotes (45). Notably, we found only a weak correlation (Spearman’s r =≈0.36) between tRNA transcript abundance and their copy number in both the EPI and MT forms, indicating that the tRNA abundance profiles in *T. cruzi* are not solely defined by gene copy numbers (Fig. 3A).

We demonstrated that tDNAs from EPI forms exhibited greater levels of open chromatin than did those from their infective forms (47), suggesting a chromatin regulatory role. Here, we investigated whether tDNA chromatin profiles are associated with tRNA expression. To this end, we used Nanopore, FAIRE-seq, and MNase-seq data sets from non-infective (EPI) and infective (MT or TCT) *T. cruzi* life forms (48, 53). The Nanopore data sets included information on DNA modifications, such as 5-methylcytosine (m^5^C) and 5-hydroxymethylcytosine (hm^5^C), which are associated with increased and decreased gene expressions, respectively (62, 63). We found that tDNAs are not localized in regions enriched in m^5^C and hm^5^C modifications compared to the shuffled control (Fig. 3B). In addition, the abundance of these DNA modifications was not associated with different tRNA expression levels in either the EPI or infective form (TCTs) (Fig. S3A and B).

MNase-seq assesses DNA regions associated with nucleosomes, providing information on nucleosome occupancy and positioning at any given locus (64). We observed lower nucleosome occupancy levels in all canonical tDNAs in both the EPI and infective forms (TCTs), indicating that tDNAs tend to be localized in nucleosome-depleted regions (NDRs) (Fig. 3C). However, tDNA loci encoding tRNA^Sec^ showed a different trend. The levels of nucleosome occupancy at the eleven tDNAs^Sec^ did not differ from those of the shuffled control, suggesting that the tDNA^Sec^ loci are localized in nucleosome-enriched regions or closed chromatin.

Next, we used the FAIRE-seq data set, which detects open chromatin, also known as transcriptionally active chromatin regions (65, 66). We obtained the abundance values of the 20 single-copy tRNA species for comparisons between their expression levels and chromatin status, mitigating the bias likely introduced by multicopy tRNA genes. The tRNAs were classified into three groups (high, medium, and low abundance) based on the tRNA abundance obtained by tRNA-seq (Fig. S3A). We found that tDNAs with high and medium abundances exhibit more open chromatin compared to those with low expression in the EPI forms, suggesting that chromatin status serves as an important regulator of tRNA expression levels. However, the open chromatin profiles of tDNA did not exhibit a distinct pattern based on the levels of tRNA abundance in *T. cruzi* infective forms (MTs), suggesting the presence of different mechanisms regulating tRNA abundance in the infective form of *T. cruzi* (Fig. 3D; Fig. S3C and Table S2). Evaluation of nucleosome occupancy levels (MNAse-seq data) retrieved similar conclusions (Fig. 3D and Fig. S3C). Notably, there is no current evidence that tRNAs are transcribed in nonproliferative/infective forms. To gain further insights into this issue, we evaluated the expression of the subunits of RNAP III, which are dedicated to tRNA transcription, in the EPI and MT forms. We detected a downregulation of multiple components of RNAP III in the MT form compared to the EPI form (Fig. 3E and Table S1F). This observation suggests lower tRNA transcriptional activity in *T. cruzi* infective forms. Consequently, additional regulatory mechanisms, independent of transcription, are likely responsible for maintaining similar tRNA abundance profiles in both EPIs and MTs.

### tRNA abundance and codon frequencies are coadapted in *T. cruzi* life forms, despite the differences in A-site ribosome occupancy

**Fig 4:**
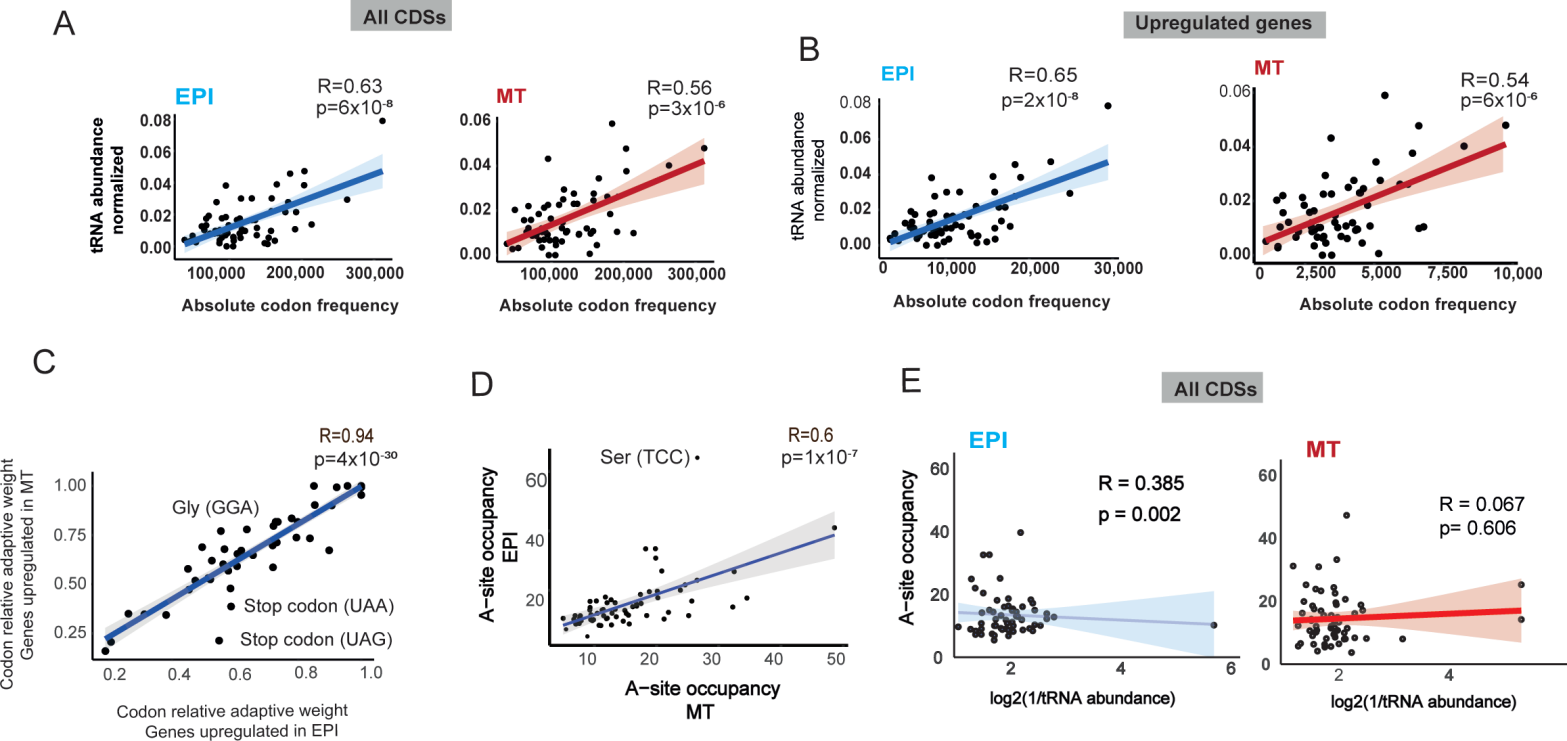
Relationship between tRNA abundance and codon usage in *T. cruzi*. (**A**) Pearson’s correlation between tRNA abundance in the EPI and MT forms with codon usage frequencies in all CDSs. (**B**) Pearson’s correlation for the codon frequency in the upregulated CDSs for the EPI (1,752 genes) and MT forms (361 genes), obtained with translatome data (Ribo-seq). (**C**) Pearson’s correlation for the relative adaptive weights of codons in the upregulated CDSs for the EPI (1,752 genes) and MT forms (361 genes), obtained from the translatome data set (Ribo-seq). The highlighted codons are at least 20% different between the indicated conditions (upregulated in EPI x upregulated in MT (**D**). Pearson’s correlation between codon occupancy at the ribosome A site in EPI and MT forms. The occupancy count for all codons at the A site was divided by 1,000 to obtain their relative frequencies. (**E**) Pearson’s correlation between A-site occupancies and tRNA abundances in EPI (left) and MT forms (right). tRNA abundance was calculated as the average of two biological replicates per life form.

It has been reported that there is a coadaptation between codon frequencies and tDNA copy number in several organisms (2, 67). However, a weak correlation between codon usage frequency and their corresponding tRNA gene (tDNAs) copy number has been observed in *T. cruzi* (37), likely due to the low correlation between tDNA copy number and their expression (Fig. 3A). Here, we investigated whether tRNA abundance, rather than copy number, is coadapted with codon frequency across all CDSs in this parasite. We found a moderate correlation between codon frequency for all CDSs and the corresponding tRNA abundance in EPIs (R = 0.63) and MT forms (R = 0.56) (Fig. 4A), indicating that tRNA abundance is coadapted with codons regardless of the parasite form. These data are consistent with Fig. 2C, where tRNA abundance profiles show only slight variations between *T. cruzi* life forms.

Next, we asked whether the codon frequency of genes differently regulated between EPIs and MTs matches their tRNA pools. The genes upregulated in EPIs showed a similar correlation with codon frequencies compared to those upregulated in MT (R = 0.65 vs R = 0.54) (Fig. 4B; Table S3A to D) and when all CDSs were compared (Fig. 4A), indicating minimal codon bias. To validate this, we analyzed whether codon bias is present among those differentially expressed CDSs. We found only a pronounced codon bias (greater than 20% usage) in stop codons (UAA and UAG) and Gly (GCC) (Fig. 4C and Table S3A), indicating that codon usage among those differentially expressed transcripts is similar in EPI and MT forms.

Despite the correlation between tRNA abundances and absolute codon frequency in both life forms, we detected a lower correlation in ribosome A-site codon occupancies between the two life forms (R = 0.6) (Fig. 4D; Table S3E). Ten out of 64 codons display statistical differences in A-site occupancy in EPIs vs MT forms (Table S3D). None of them are associated with tRNA abundance differences previously detected (Fig. 2C). This suggests that, although codon usage is similar in upregulated genes from infective and non-infective forms, certain codons may be translated more efficiently—or be more prone to ribosomal pausing—depending on the *T. cruzi* life form. The differences between life forms become evident when tRNA abundances are directly compared to ribosome A-site occupancies, revealing a complete loss of correlation in MT forms (R = 0.07) (Fig. 4E). These findings suggest that additional regulatory layers—beyond tRNA abundance—may contribute to the translational differences observed between EPI and MT forms.

### tRNA abundance is associated with translated transcript levels in EPI and MT forms

**Fig 5:**
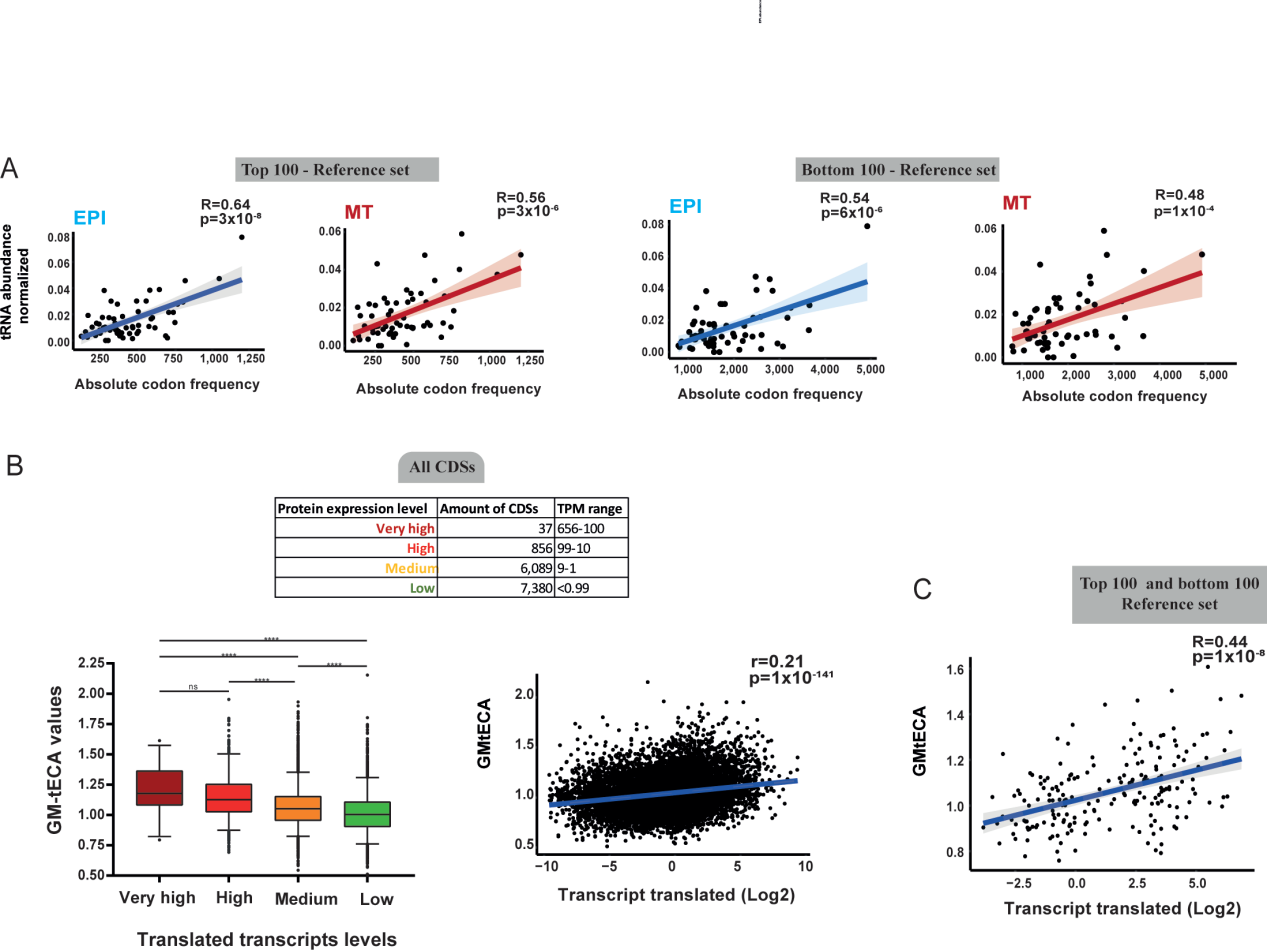
Relationship between tRNA abundance and translated transcript levels in *T. cruzi*. (**A**) Pearson’s correlation between tRNA abundance in the EPI and MT forms with codon frequencies of higher (top 100) and lower (bottom 100) translated transcripts. Shading—95% CI. (**B**) Association between tRNA abundance and translated transcript expression levels from 14,363 CDSs. The table shows translated transcript expression levels (very high, high, medium, and low) based on TPM values from Ribo-seq data in EPI forms. The box plot displays GM-tECA values, which is the geometric mean of tRNA renormalized abundances for each codon. The 5th to 95th percentiles are represented by black points. One-way ANOVA was used to test for significance. Values with *P* ≤ 0.05 were considered statistically significant. ns = not significant. (**C**) Pearson’s correlation between transcript translated levels from reference set (top 100 and bottom 100) and tRNA abundance, as quantified by GM-tECA values. tRNA abundance was calculated as the average of two biological replicates per life form.

Given the differential codon at the ribosomal A site in EPI and MT forms, we investigated whether highly translated transcripts are preferentially enriched in codons that match the most abundant tRNAs in *T. cruzi*. Thus, we evaluated the association between codon frequency and tRNA abundance for the more abundant and less abundant translated transcripts from a reference set (Tables S1E; S3A and D), which were created to avoid multiple-copy genes and transcripts that exhibited large differences (fold change log2 ≥2) in expression between life forms. We found that the correlation was greater for codons from the highly abundant transcripts (top 100) than for those from the less abundant transcripts (bottom 100) in both EPIs and MTs (Fig. 5A), suggesting that CDSs with poorly adapted codons to tRNA abundance may exhibit lower expression levels.

To validate this globally, we developed a metric based on the geometric mean of the abundance of all tRNAs associated with decoding the codons of a given CDS. As a result, higher GM-tECA values indicate CDSs that are well-coadapted to their tRNA pool levels. To do that, we applied the tRNA abundance values for each corresponding codon triplet and calculated the geometric mean of these values for all CDSs present in the *T. cruzi* genome, excluding CDSs without protein expression values. Translated transcript expression levels from all CDSs were classified as very high, high, medium, and low according to their TPM values. Therefore, we observed that CDSs expressed in EPI or MT forms, with high expression levels, show greater GM-tECA values compared to those with lower expression (Fig. 5B; Fig. S4 and Table S4). This association is clearer when analyzing the top and bottom 100 genes of the reference set (Fig. 5C and Table S4). Despite this, we find no correlation between tRNA abundance and translational efficiency (TE) (Fig. S5; Table S3F), indicating that tRNA abundance contributes to determining translated transcript expression levels; however, TE is predominantly controlled by other layers of translation regulation.

### Base pairing modes between codons and anticodons are linked to translated transcript levels

**Fig 6:**
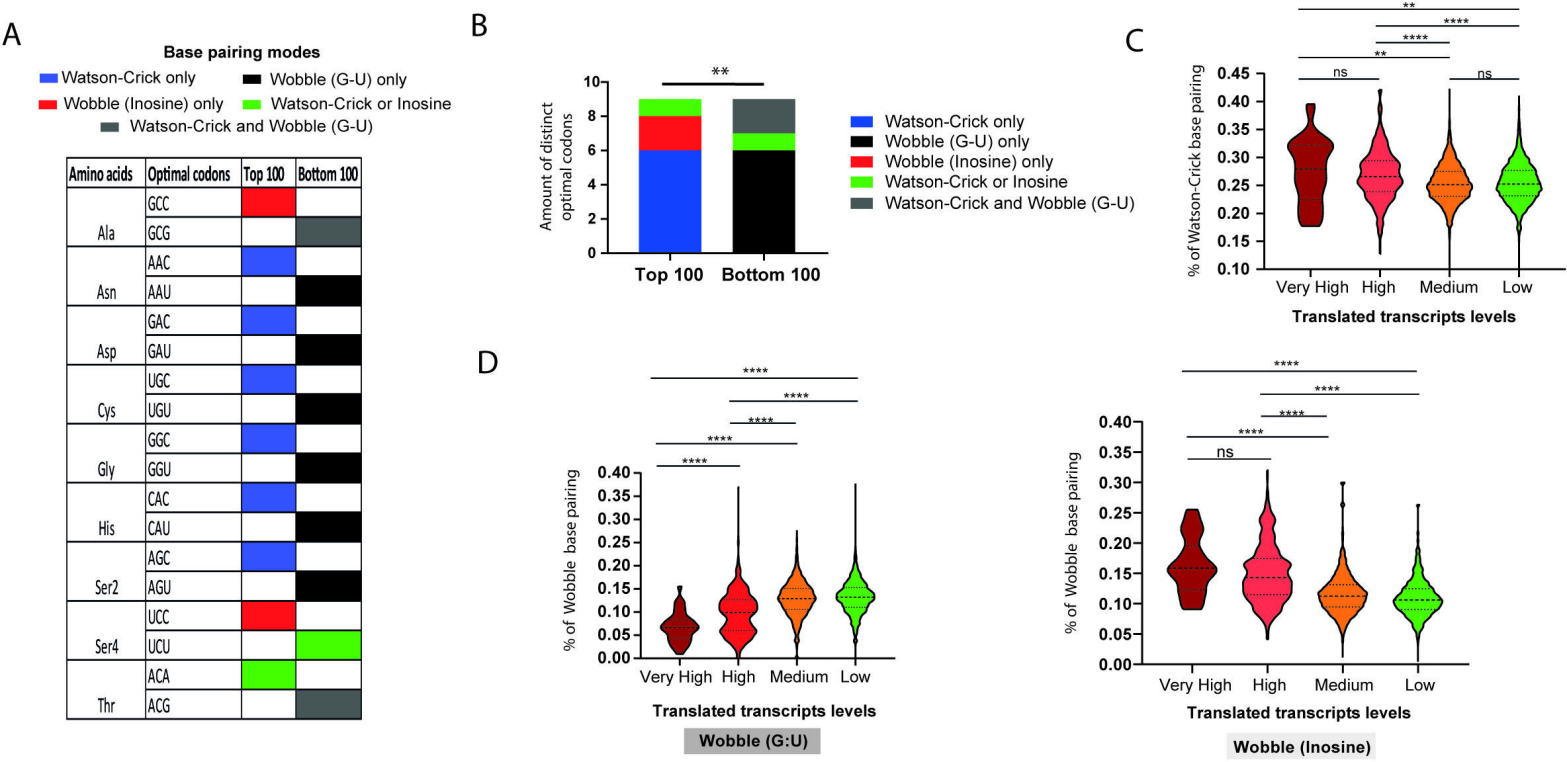
Anticodon:codon base pairing modes correlate with translated transcript levels. (**A**) Distinct optimal codons between the highest 100 (top 100) and lowest 100 (bottom 100) translated transcripts in the EPI and MT forms from the reference set. Optimal codons were defined as the most frequently used codon that encodes either a specific amino acid as described at (Sharp and Li 1987b) (Sharp and Li 1987b). See Table S3A for a complete list of optimal codons. (**B**) Distribution of the anticodon:codon base pairing modes [(i) Watson–Crick only, (ii) wobble (G–U) only, (iii) wobble (inosine) only, (iv) Watson–Crick and wobble (inosine), and (v) Watson–Crick and wobble (G:U)] presented in distinct optimal codons on the top 100 and bottom 100 translated transcripts. Chi-square*-P* value ≤ 0.01 (**) was considered. (**C**) Violin plot showing the percentage of Watson-Crick base pairing in all CDSs and the translated transcript expression levels in EPI forms. (**D**) Association between the percentage of Wobble base pairing (G:U or inosine) in all CDSs with translated transcript levels in EPI forms. Very high expression levels correspond to CDSs with TPM values ranging from 656 to 100 (37 transcripts), high expression levels range from 99 to 100 (856 transcripts), medium expression ranges from 9 to 1 (6,089 transcripts), and low expression is defined as <0.99 (7,380 transcripts). One-way ANOVA was used to test for significance. Values with *P* ≤ 0.05 were considered statistically significant. ns = non-significant.

To further understand the observation that highly expressed transcripts (top 100) are more strongly correlated with tRNA abundance than are less expressed transcripts (bottom 100), we performed a detailed analysis of the codon composition of our reference set, focusing on the top 100 and bottom 100 transcripts mentioned in Fig. 5A. We identified 46 tRNA anticodons to 62 codons in the *T. cruzi*, implying that some tRNA transcripts must recognize more than one codon via wobble base pairing (Fig. 1D). Intriguingly, we found nine distinct optimal codons between the top 100 or bottom 100 transcripts (Fig. 6A and Table S3A). With the exception of Ala (GCC/GCG), all of the remaining synonymous codons are recognized by the same set of tRNA species. Strikingly, six (out of nine) optimal codons from the top 100 abundant transcripts interact via Watson–Crick base pairing only, while six (out of nine) optimal codons from the bottom 100 form only wobble base pairing (Fig. 6B). This observation indicates that the anticodon:codon base pairing mode may be associated with the expression level of the translated transcripts in *T. cruzi*.

Thus, we examined whether the association between anticodon:codon pairing modes and translated transcript levels holds for the full repertoire of translated transcripts in EPI and MT forms. Thus, we calculated the percentage of codons exhibiting either Wobble or Watson-Crick base pairing for each mRNA. Globally, translated mRNAs with higher expression levels tend to be more enriched in codons with Watson-Crick base pairing compared to transcripts with lower expression levels, in both infective and noninfective forms (Fig. 6C and D; Fig. S6A and Table S4). In contrast, for Wobble (G:U) base pairing, the trend is reversed: lower expression of translated transcripts is associated with a higher enrichment of Wobble (G:U), while higher translated transcript expression shows reduced enrichment of Wobble (G:U). Similar results were also observed for genes from our reference set (Fig. S6A). Additionally, we found that codons exhibiting both Watson-Crick and Wobble (G:U) pairings are not associated with translated transcripts expression (Fig. S6B), emphasizing that only codons that form exclusively either Watson-Crick or Wobble (G:U) pairings might be important for directing protein expression levels. Furthermore, we found that mRNAs enriched with codons containing inosine base pairings are more associated with highly expressed translated mRNAs than with those that are lesser expressed, similar to what was observed with codons enriched in Watson-Crick base pairing (Fig. 6C and D Fig. S6A to S6B). Overall, our results suggest that anticodon:codon base pairing modes display a significant regulatory layer in protein expressions in both infective and noninfective forms of *T. cruzi*.

## DISCUSSION

Codon usage bias has been proposed to be a significant factor directing transcript and protein expression levels in trypanosomes (6, 36). Codon frequency is linked to the abundance of tRNA pools in some species, but measuring tRNA expression levels has been only a recent advancement (45). Here, we sequenced tRNAs in two different developmental forms of *T. cruzi*, which enabled direct comparisons between tRNA abundance and codon usage biases. We observed a stronger correlation between codon usage and tRNA abundance than between the copy number of tRNA genes (Fig. 4A), highlighting the importance of obtaining actual tRNA expression profiles to analyze the relationship between codon bias and tRNA supply. Surprisingly, tRNA abundance and codon usage preference are very similar in EPI and MT forms (Fig. 4C), although these two forms have distinct gene expression profiles (33, 35) and A-site ribosome occupancies (33, 35). We developed a pipeline to measure the adaptation of CDSs to their corresponding tRNA abundance. In general, we found that translated transcript expression levels in infective and noninfective forms of *T. cruzi* correlate with tRNA abundance and anticodon:codon base pairing (Fig. 5 and 6).

The transcription of tRNAs relies on RNA Pol III and interactions between transcription factors and promoters within tRNA loci. This process is facilitated by the depletion of nucleosomes at these loci (68). Consequently, these results indicate that the chromatin status can influence tRNA transcription. Notably, we found a relationship, exclusive to EPI forms, between tDNA open chromatin levels and tRNA expression (Fig. 3D). In trypomastigote forms (both TCTs and MTs), tRNA expression appears to be independent of chromatin status (Fig. 3D), suggesting that other regulatory mechanisms maintain the tRNA abundance profiles in MTs similar to those in the EPI form. A possible explanation for the similar tRNA abundance profiles in the MT and EPI forms is that *de novo* tRNA synthesis is not active in the MT forms, resulting in maintenance of the same tRNA profiles as in the EPI form (Fig. 2C). The low amount of mature tRNAs (per parasite) in MT forms (47), alongside a downregulation of the RNAP III machinery in these forms (Fig. 3E), suggests that tRNA synthesis is not active in MTs, while nascent expression via RNA Pol III has not yet been assessed in *T. cruzi*. Pol III RNA expression can also be arrested by Maf1, which was described in *T. brucei* to inhibit RNAP III transcription in procyclic forms (69). Since Maf1 is known to bind to RNAP III under stress conditions in some organisms (68), we hypothesized that a similar mechanism may also play a role during *T. cruzi* differentiation. Although we observed similar results using either TCTs or MTs—both non-proliferative and infective forms—it is important to emphasize that they are distinct life forms and may exhibit different regulatory programs, particularly regarding tRNA expression and regulation. In addition, differences observed between epimastigotes and metacyclic trypomastigotes may reflect both developmental and proliferative state differences.

The observed correlation between tRNA abundance and codon bias may reflect not only transcriptional regulation but also differences in tRNA stability. Post-transcriptional modifications can enhance or reduce tRNA stability, potentially affecting their abundance across life forms. Although tRNAs are generally considered highly stable molecules, various stress conditions are known to alter their half-lives in several organisms (70, 71). In trypanosomes, for instance, tRNA^Tyr^ exhibits an unusually short half-life (72) and nutritional stress has been associated with increased levels of tRNA-derived fragments (73), suggesting active degradation under such conditions. Therefore, future investigations into tRNA half-lives, their degradation pathways, and associated modifications in *T. cruzi* could provide further insights into their impact on protein expression. Many bioinformatics methods are used to measure the degree of coadaptation between CDS expression and the tRNA pool (2). The tRNA adaptation index (tAI) and its derivatives (such as gtAI (74)) are one such approach and are widely used in many organisms with multiple tDNA copy numbers as a higher copy number generally corresponds to higher tRNA expression compared to those with fewer copies (45, 67, 74). However, in some organisms, such as *T. cruzi*, its use is not recommended due to the few tDNA copy numbers (Fig. 1A) and the weak correlation between tDNA copy number and tRNA transcript expression (Fig. 3A). We found that tRNA abundance contributes to determining CDS expression levels in both infective and noninfective forms of *T. cruzi*, as determined by our GM-tECA approach combined with translatome data. It differs from other existing metrics, which rely on tRNA gene copy numbers, anticodon:codon pairing modes, and computational decoding predictions to estimate protein expression. In other words, GM-tECA is an empirical method specifically designed to evaluate how tRNA abundance alone may influence protein expression levels, when integrated with large-scale data sets such as Ribo-seq or quantitative proteomics. GM-tECA was not intended to replace existing tRNA metrics, but rather to provide a simple and intuitive summary of how well a transcript’s codon usage aligns with the tRNA abundance landscape. We believe that GM-tECA can be a powerful exploratory tool to identify potential CDSs affected by tRNA dysregulation across various cellular models. This is particularly relevant in multiple cancer contexts, where abundance alteration in some tRNA species has been associated with changes in protein synthesis that contribute to cancer development and progression (75–77).

Trypanosomatids have fewer copies of tRNA genes (fewer isodecoders) (Fig. 1A) than vertebrates (60, 78), suggesting that these genes may be under selective pressure to avoid deleterious mutations. Here, we detected 46 anticodons for 62 codons (Fig. 1D), which contrasts with findings in humans, where there are 57 anticodons for 61 codons (79, 80). This indicates that the parasite needs to employ more strategies to recognize the entire codon repertoire of the genome, such as employing more modifications in anticodons and anticodon:codon wobble pairing. In this sense, we found that *T. cruzi* may take advantage of another layer of gene expression regulation based on anticodon:codon pairing modes. We found that the most abundant genes from each life form predominantly use codons that utilize either Watson–Crick or inosine base pairing (Fig. 6C and D). In contrast, the less abundant transcripts exhibited higher frequencies of wobble (G:U) pairing in anticodon interactions. In *Plasmodium falciparum*, it has been reported that wobble G:U pairs are associated with reduced translational efficiency (81). Such skewed synonymous codon usage can be a common strategy employed by parasites to optimize translation.

Regarding inosine modifications within the tRNA anticodon region, overexpression of A34-to-I34 modifying enzymes (ADAT2/3) in EPIs led to altered inosine levels and changes in the expression of surface mucins (TcMUSG-L vs TcMUSG-S), which differs in threonine codon usage (82). Here, we did not observe differences in I34 levels—either globally or in tRNA^Thr(AGT)^—between EPIs and MTs, suggesting that the life stage-specific expression of mucins may not be driven by this modification. Still, tRNA modifications remain a promising layer of post-transcriptional regulation, warranting further investigation.

Considering that the trypanosome mitochondrial genome lacks tRNA genes, mitochondrial translation relies on nuclear-encoded tRNAs (83). Given the correlation we observed between tRNA abundance and nuclear codon bias, one might expect similar constraints in mitochondrial genes. However, extensive RNA editing in mitochondrial transcripts (84) and lack of data on mitochondrial tRNA levels preclude a clear analysis of codon bias in mitochondrial genes and remain a subject for future investigation.

Finally, our data distinguish two axes associated with translational regulation in *T. cruzi*. First, at the genome-wide level, we observed a strong correlation between codon usage and tRNA availability, highlighting a finely tuned codon-anticodon co-adaptation. This coordination aligns with transcript abundance but does not fully account for translational efficiency. These genome-wide features are consistent across both life forms, which display similar codon usage and tRNA pools—though correlations are slightly lower in the MT form. Second, we observed notable differences in A-site ribosome occupancy between epimastigote and metacyclic trypomastigote forms (R = 0.6; Fig. 4D), suggesting that translation efficiency is modulated by differential codon occupancy at the ribosome A site. This indicates that while the conserved tRNA pool represents a general translational adaptation at the genome level, the life stage-specific differences in A-site codon occupancy likely reflect additional layers of regulation. These may include changes in ribosome dynamics, mRNA secondary structure, tRNA modifications, or the activity of RNA-binding proteins and other translational regulators that influence ribosome activity (85–87). By clearly distinguishing these two dimensions—global codon–tRNA adaptation and stage-specific translational modulation—we clarify that the limited differences in tRNA composition are not inconsistent with the observed changes in translation efficiency between EPI and MT forms.

In brief, we propose that *T. cruzi* protein expression in EPIs and MTs may be affected by a combination of codon usage bias, tRNA abundance, and anticodon:codon pairing mode. Subsequent studies should explore the potential impact of perturbed tRNA biology on parasite viability, thereby offering promising avenues for novel disease-control strategies, particularly in the realm of tRNA-based therapeutics.

## Data Availability

The tRNA-seq, MNAse-seq, and FAIRE-seq data can be accessed at the Sequence Read Archive (SRA) (https://www.ncbi.nlm.nih.gov/sra) under the following accession numbers: PRJNA1124437, PRJNA665060, and PRJNA763084. Oxford Nanopore sequencing data sets are available at (28).

## SUPPLEMENTAL MATERIAL

Supplemental figures mbio.01622-25-s0001.pdf Fig. S1 to S6.

Table S1 mbio.01622-25-s0002.xlsx Characterization of tDNA and tRNA pools in *T. cruzi* Dm28c.

Table S2 mbio.01622-25-s0003.xlsx Abundance of single-copy tRNAs and their levels of open (FAIRE-seq) and closed (MNase-seq) chromatin in the EPI and MT forms.

Table S3 mbio.01622-25-s0004.xlsx Codon usage in the *T. cruzi* genome.

Table S4 mbio.01622-25-s0005.xlsx Translated transcript expression values, GM-tECA, and percentage of anticodon:codon base pairing for all CDSs in T*. cruzi*.

ASM does not own the copyrights to Supplemental Material that may be linked to, or accessed through, an article. The authors have granted ASM a non-exclusive, world-wide license to publish the Supplemental Material files. Please contact the corresponding author directly for reuse.
