# Supplementary material for: Integrating tRNA gene epigenomics and expression with codon usage unravels an intricate connection with translatome dynamics in Trypanosoma cruzi
Source: mBio. 2025 Aug 11;16(9):e01622-25. doi: 10.1128/mbio.01622-25 (PMC12421993; doi:10.1128/mbio.01622-25)
Supplement: Supplemental figures — Fig. S1 to S6. [file mbio.01622-25-s0001.pdf]

Supplementary Information - Legends

Fig. S1. Quantity of reads and sequencing data quality for tRNA-seq performed in the EPI and MT forms. **A)** Number of reads sequenced, filtered and uniquely mapped on tDNAs from *T. cruzi* Dm28c. **B)** Quality of base pair sequences, assessed by Phred scores, of a representative sample (MT R1). **C)** PCA plot of tRNA-seq-normalized samples with the varianceStabilizingTransformation tool from the DESeq2 package. R1= replicate 1; R2= replicate 2. **D)** Metagene analysis of the normalized sequence coverage of tRNA isotypes.

Fig. S2. tRNA abundance in *T. cruzi* life forms. **A)** Scatter plot showing Pearson's correlation between tRNA abundance (proportion of mapped reads) in two biological replicates (R1 and R2) in EPI or MT forms. Gray shading: 95% confidence interval. **B)** Heatmap containing the tRNA abundance in EPI and MT forms. The experiments were performed in biological duplicates (R1 or R2). **C)** Isoacceptor tRNA expression analysis. Scatter plot of fold changes of tRNA anticodon expression (Log2) in EPIs compared to MTs. The significance of tRNA differential expression was determined using the DESeq2 algorithm considering a p-adjust value of ≤ 0.05. **D)** Abundance of tRNAs found within clusters and alone (single). Twenty-single copy tRNAs were considered: 15 in clusters and 5 alone. No statistical difference was found (t-test).

Fig. S3. Abundance of twenty tRNA transcripts of a single copy in *T. cruzi* and their association with tDNA modifications. **A)** Bar charts depicting the seven tRNAs with the highest abundance, the seven with medium expression, and the remaining six with the lowest abundance in either the EPI or MT form. **B)** Relationships between tRNA abundance levels (high, medium and low) and hm^5C or m^5C modification levels in twenty single-copy tDNAs in the EPI and MT forms. Statistical significance tests were performed with the Wilcoxon–Mann–Whitney test. (ns = not significant). C) Pearson's correlation between tRNA abundance and chromatin accessibility levels—RPGC values from FAIRE-seq (open chromatin) and FPKM values from MNase-seq (closed chromatin)—in EPI and MT forms.

Fig. S4. Association between tRNA abundance and translated transcripts expression levels. The table shows translated transcripts levels (very high, high, medium, and low) based on TPM values from Ribo-seq data in MT forms. The box plot displays GM-tECA values, which is the geometric mean of tRNA renormalized abundances for each

corresponding codon. The 5th and 95th percentiles are represented by black points. One-way ANOVA was used to test for significance. Values with $p \leq 0.05$ were considered statistically significant.

Fig. S5. Spearman's correlation between translational efficiency (TE) of CDSs from the reference set list (top 100 and bottom 100) and tRNA abundance as quantified by GM-tECA values in EPI and MT forms.

Fig. S6. Association between translated transcripts levels and anticodon:codon base pairing. **A)** Correlation between the percentage of Watson-Crick or Wobble (G:U or Inosine) base pairing and in all CDSs with translated transcripts expression levels values in MT forms. High expression levels correspond to CDSs with TPM values ranging from 158 to 10 (230 genes), medium expression ranges from 9 to 1 (2,813 genes), and low expression is defined as <0.99 (10,171 genes). **B)** Relationship between the percentage of base pairing modes (Wobble (G:U) only, Watson-Crick only, Wobble (Inosine) only and Watson-Crick and Wobble (G:U)) with translated transcripts expression from reference set in EPI and MT forms. One-way ANOVA was used to test for significance. Values with $p \leq 0.05$ were considered statistically significant. ns= not significant. **C)** Pearson's Correlation between tRNA abundance of CDSs from the reference set list (Top 100 and bottom 100) and tRNA abundance based on GM-tECA values in EPI.

Supplementary Tables Legends

Table S1. Characterization of tDNA and tRNA pools in *T. cruzi* Dm28c. **A)** Genomic coordinates of tDNAs, gene IDs, sense strands and copy numbers. **B)** Identification of tRNA anticodons and their base pairing types with corresponding codons in the *T. cruzi* genome**. C)** tRNA abundance of replicates (R1 and R2) and their averages in both the EPI and MT forms. **D)** DESeq results of tRNA abundance in EPIs compared to the MTs. **E)** Correlations between tRNA abundance and base pairing types with their corresponding codons. **F)** BLAST and HMMER results containing the best hit of the RNAP III subunit candidates present in the *T. cruzi* genome.

Table S2. Abundance of single-copy tRNAs and their levels of open (FAIRE-seq) and closed (MNase-seq) chromatin in the EPI and MT forms.

Table S3. Codon usage in the *T. cruzi* genome. **A)** Codon frequencies and codon relative adaptive weight for all CDSs, upregulated genes, top 100 and bottom 100 genes. **B)** TPM values for all genes in the EPI and MT forms obtained from the translatome data (Ribo-seq). **C)** DESeq2 results from Ribo-seq data performed in the EPI and MT forms.

D) Gene IDs of the reference set (top 100 and bottom 100) and genes upregulated in the EPI and MT forms. **E)** Codon occupancy at the ribosome A site for all codons in EPI and MT forms. **F)** TE values from EPI and MT forms.

Table S4. Values containing the translated transcripts expression values (TPM), GM-tECA and percentage of anticodon:codon base pairing for all CDSs in *T. cruzi.*

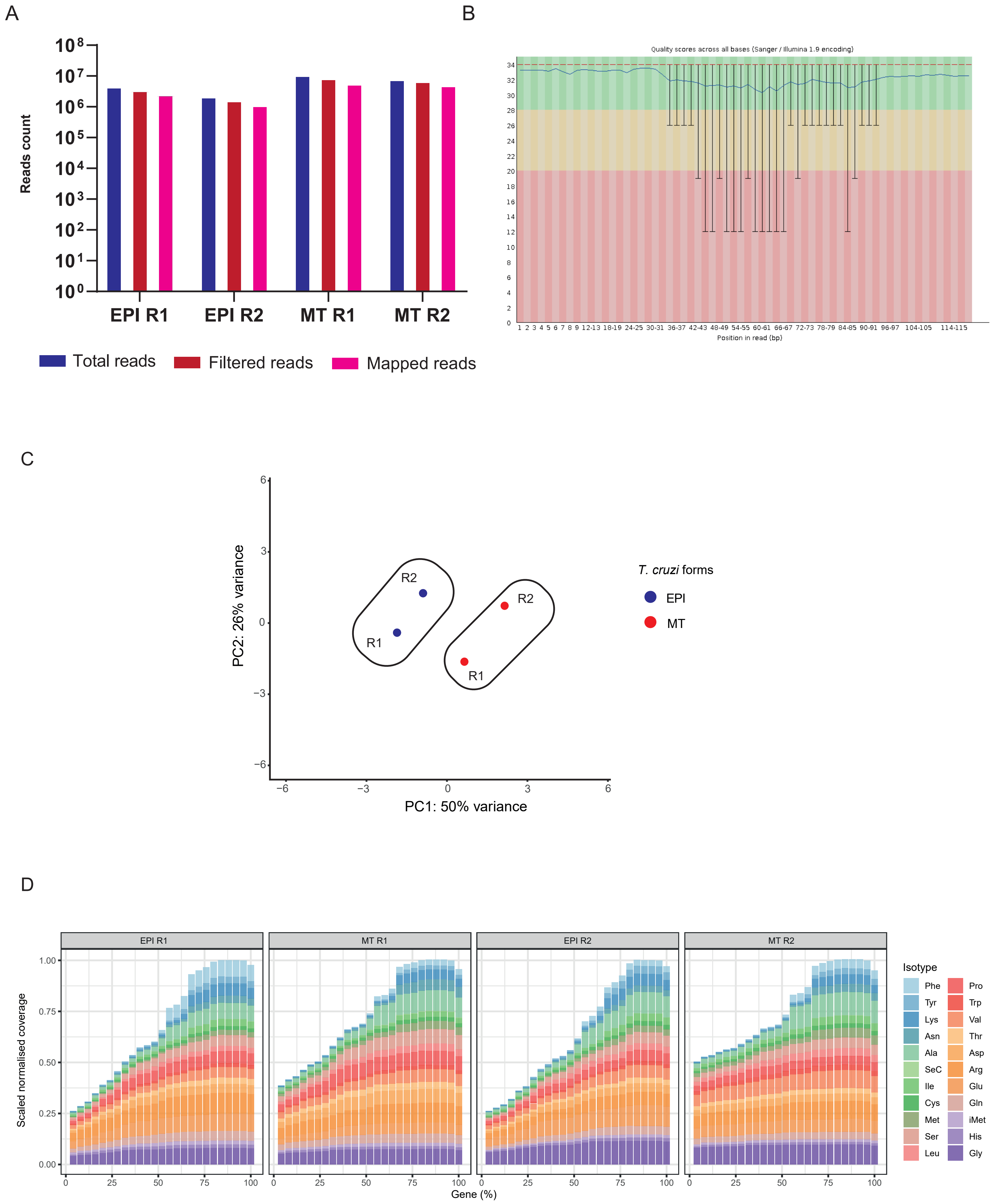
A
B
C
D
Reads count
EPI R1
EPI R2
MT R1
MT R2
Total reads
Filtered reads
Mapped reads
Quality scores across all bases (Sanger / Illumina 1.9 encoding)
Position in read (bp)
PC2: 26% variance
PC1: 50% variance
T. cruzi forms
EPI
MT
R1
R2
Scaled normalised coverage
Gene (%)
Isotype
Phe
Tyr
Lys
Asn
Ala
SeC
Ile
Cys
Met
Ser
Leu
Pro
Trp
Val
Thr
Asp
Arg
Glu
Gln
iMet
His
Gly

A

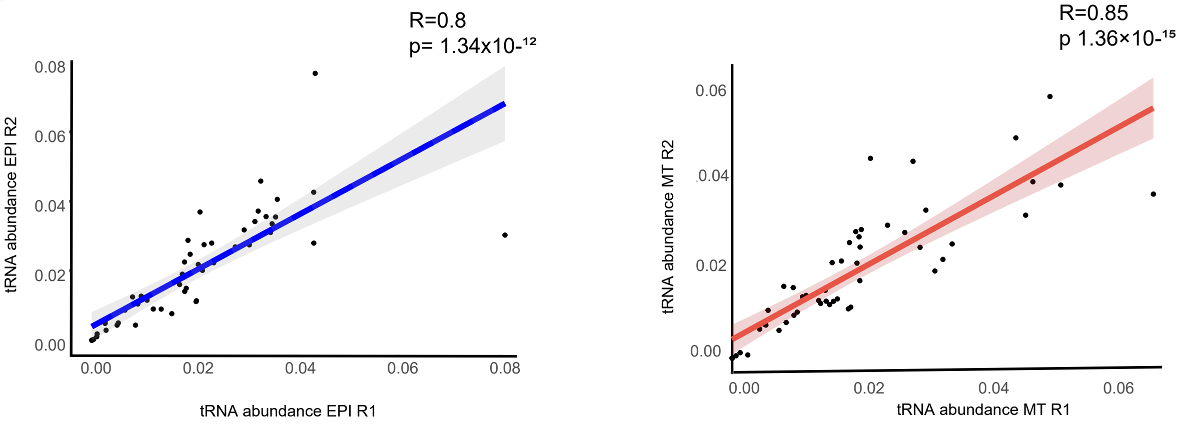
R=0.8
p= 1.34x10-¹²
tRNA abundance EPI R2
tRNA abundance EPI R1
0.00
0.02
0.04
0.06
0.08
R=0.85
p 1.36×10-¹⁵
tRNA abundance MT R2
tRNA abundance MT R1
0.00
0.02
0.04
0.06

B

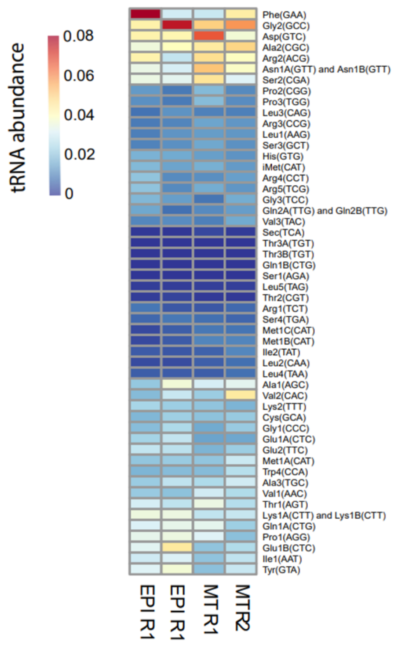
tRNA abundance
0.08
0.06
0.04
0.02
0
Phe(GAA)
Gly2(GCC)
Asp(GTC)
Ala2(CGC)
Arg2(ACG)
Asn1A(GTT) and Asn1B(GTT)
Ser2(CGA)
Pro2(CGG)
Pro3(TGG)
Leu3(CAG)
Arg3(CCG)
Leu1(AAG)
Ser3(GCT)
His(GTG)
iMet(CAT)
Arg4(CCT)
Arg5(TCG)
Gly3(TCC)
Gln2A(TTG) and Gln2B(TTG)
Val3(TAC)
Sec(TCA)
Thr3A(TGT)
Thr3B(TGT)
Gln1B(CTG)
Ser1(AGA)
Leu5(TAG)
Thr2(CGT)
Arg1(TCT)
Ser4(TGA)
Met1C(CAT)
Met1B(CAT)
Ile2(TAT)
Leu2(CAA)
Leu4(TAA)
Ala1(AGC)
Val2(CAC)
Lys2(TTT)
Cys(GCA)
Gly1(CCC)
Glu1A(CTC)
Glu2(TTC)
Met1A(CAT)
Trp4(CCA)
Ala3(TGC)
Val1(AAC)
Thr1(AGT)
Lys1A(CTT) and Lys1B(CTT)
Gln1A(CTG)
Pro1(AGG)
Glu1B(CTC)
Ile1(AAT)
Tyr(GTA)
EPI R1
EPI R1
MT R1
MTR2

C

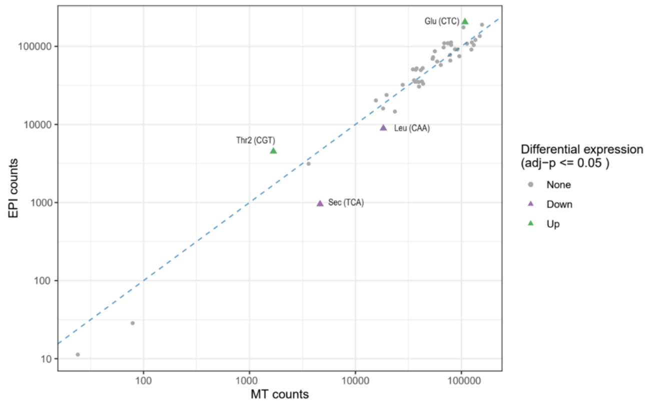
Glu (CTC)
Thr2 (CGT)
Leu (CAA)
Sec (TCA)
EPI counts
MT counts
10
100
1000
10000
100000
Differential expression
(adj−p <= 0.05)
None
Down
Up

D

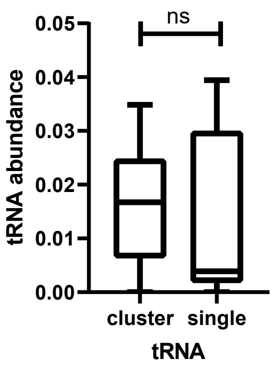
ns
tRNA abundance
0.00
0.01
0.02
0.03
0.04
0.05
cluster
single
tRNA

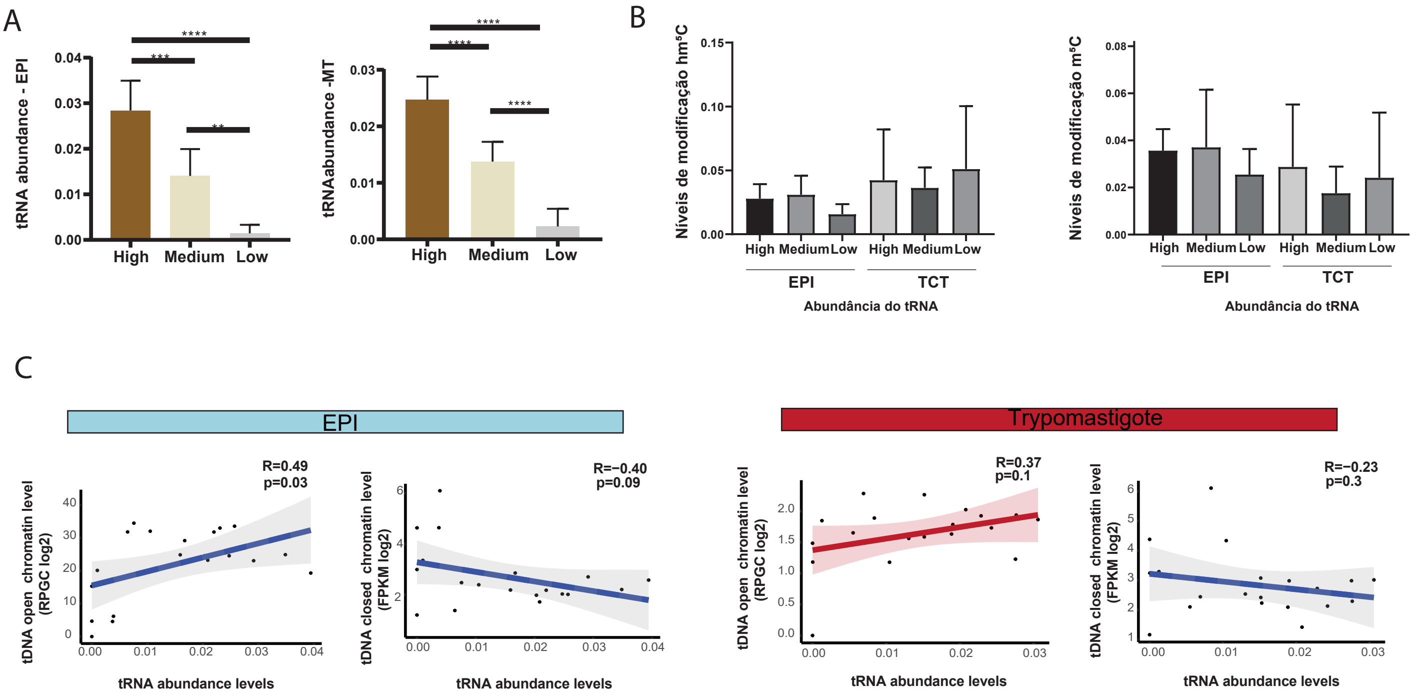

A
tRNA abundance - EPI
tRNAabundance -MT
High
Medium
Low
B
Niveis de modificação hm⁵C
Niveis de modificação m⁵C
EPI
TCT
Abundância do tRNA
C
EPI
Trypomastigote
R=0.49
p=0.03
R=−0.40
p=0.09
R=0.37
p=0.1
R=−0.23
p=0.3
tDNA open chromatin level (RPGC log2)
tDNA closed chromatin level (FPKM log2)
tRNA abundance levels

All CDSs

Protein expression level	Amount of CDSs	TPM range
Very high	Absent	Absent
High	230	158-10
Medium	2,813	9-1
Low	10,171	<0.99

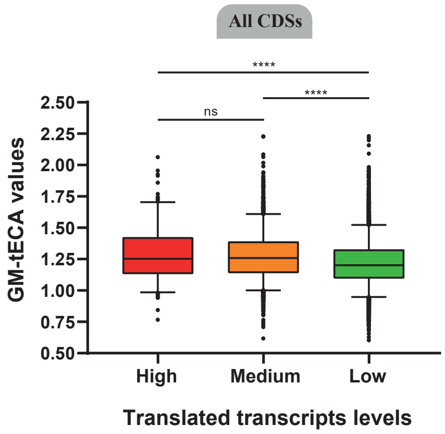

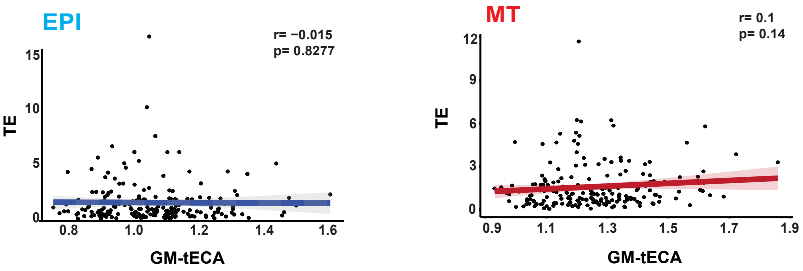

EPI
r= −0.015
p= 0.8277
TE
GM-tECA
0
5
10
15
0.8
1.0
1.2
1.4
1.6
MT
r= 0.1
p= 0.14
TE
GM-tECA
0
3
6
9
12
0.9
1.1
1.3
1.5
1.7
1.9

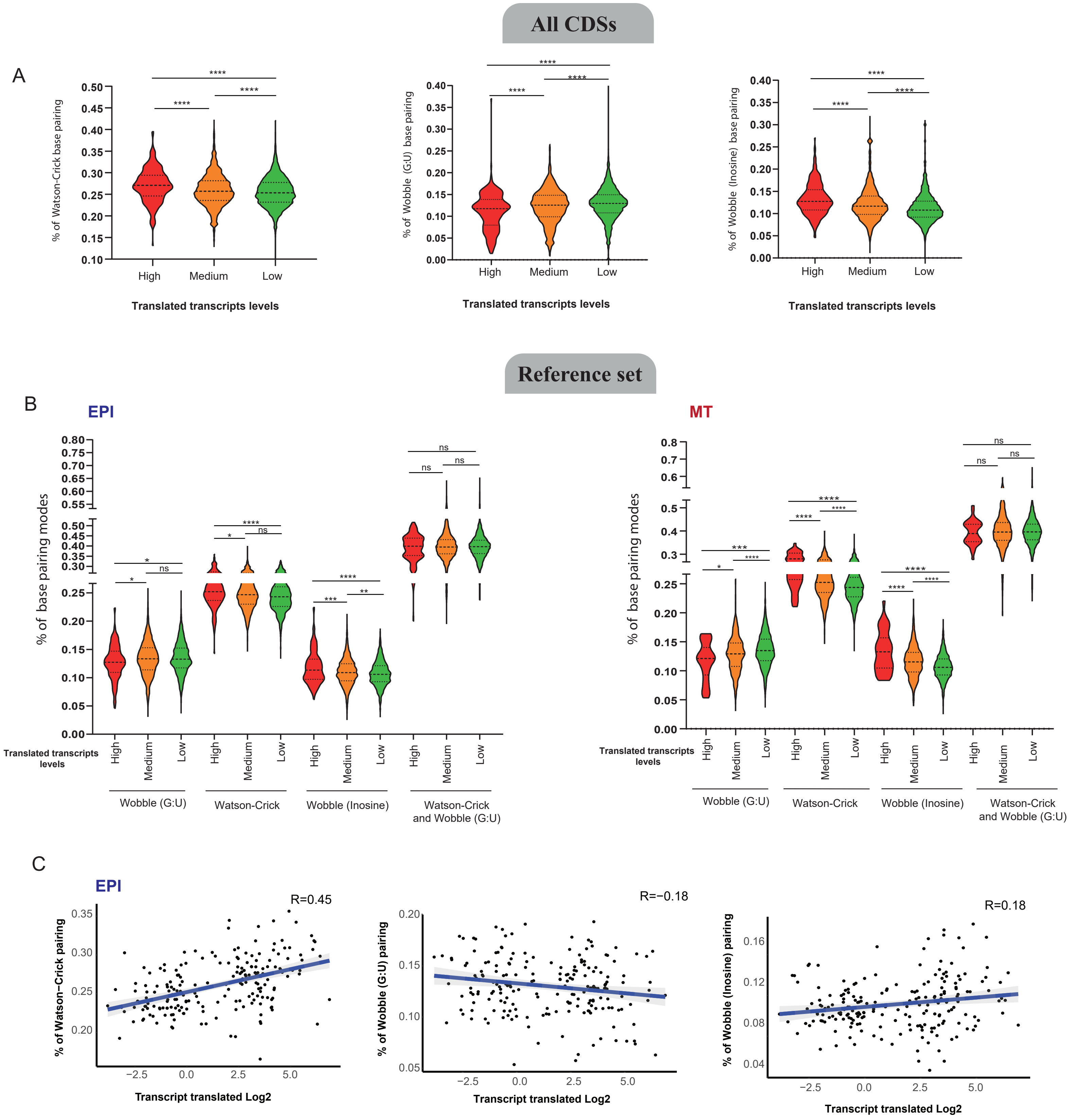

All CDSs
A
% of Watson-Crick base pairing
% of Wobble (G:U) base pairing
% of Wobble (Inosine) base pairing
High
Medium
Low
Translated transcripts levels
****
Reference set
B
EPI
MT
% of base pairing modes
Translated transcripts levels
Wobble (G:U)
Watson-Crick
Wobble (Inosine)
Watson-Crick and Wobble (G:U)
ns
*
**
***
C
EPI
R=0.45
R=−0.18
R=0.18
% of Watson−Crick pairing
% of Wobble (G:U) pairing
% of Wobble (Inosine) pairing
Transcript translated Log2
